# Design of an artificial intelligence model to screen spontaneous speech to detect Alzheimer’s Disease

**DOI:** 10.1371/journal.pdig.0001444

**Published:** 2026-06-25

**Authors:** Kenneth M. Madden, Boris Feldman, Daniel Jimenez, Mandeep Khokhar, Qihan Li, Minh Nguyen, Jian Zhu

**Affiliations:** 1 Gerontology and Diabetes Research Laboratory, Division of Geriatric Medicine, Department of Medicine, University of British Columbia, Vancouver, British Columbia, Canada; 2 Centre for Aging SMART, University of British Columbia, Vancouver, British Columbia, Canada; 3 Edwin S. H. Leong Centre for Healthy Aging, University of British Columbia, Vancouver, British Columbia, Canada; 4 UBC Data Science Program, University of British Columbia, Vancouver, British Columbia, Canada; National Research Center for Rehabilitation Technical Aids, CHINA

## Abstract

There is a shortage of physicians trained in the specialized care of Alzheimer’s disease (AD). One possible solution is to use machine learning (ML)/artificial intelligence (AI) techniques to screen for the effects of AD on speech patterns, a technique that has the potential for automation. Our objective was to evaluate the ability of various language processing/artificial intelligence (AI) techniques to classify subjects into AD and controls. Our study used the Pitt dataset (n = 549), from DementiaBank, a shared dataset of audio files of spontaneous speech using the standardized Cookie Theft Picture Description task. The dataset was divided into training (n = 337), development (n = 115) and test (n = 97) datasets. A series of language processing techniques were tested on their ability to classify subjects into AD and controls, including classical (Tfidf, Term Frequency-Inverse Document Frequency), hybrid (Tfidf + text embeddings), neural classification and large language models. The hybrid model (Tfidf + text embeddings) was the best performing one, with accuracy and recall of 0.92 and 0.93 respectively on the test dataset. However, when tested on an independently collected validation dataset it only achieved 0.70 accuracy and 0.44 recall, performing well in identifying controls but missing some true AD cases. The model also demonstrated similar performance in identifying controls in a dataset that included a high proportion of diverse cognitive conditions, similar to referrals to a specialized memory clinic. AI techniques have demonstrated some utility for the classification of spontaneous speech audio files into those with AD and controls although these techniques could benefit from having enlarged datasets.

## Author summary

In the early stages of dementia, there are subtle changes in speech–some have suggested that machine learning/artificial intelligence methods might be a way to automate screening for dementia in order to address the shortage of physicians trained in the specialized care of older adults. We evaluated the ability of various language processing/artificial intelligence techniques to classify subjects into Alzheimer’s Disease and controls. Our hybrid model (Term Frequency-Inverse Document Frequency + Large Language Model text embeddings) was the best performer, but when tested on a separate validation dataset it was much better at identifying controls than detecting true AD cases. Most previous work in this field has selected and tested a single machine learning model chosen ahead of time as opposed to a more organized pipeline for systematic model selection. Previous work has also relied on data partitions (that is splitting the data into test sets) as opposed to using a truly independent validation dataset. Our approach has shown that our hybrid model approach is the best performer, but when tested on an independent set of recordings this approach may have more of a role in triaging patients, as opposed to providing a reliable clinical diagnosis.

## Introduction

The most common etiology of dementia is Alzheimer’s disease, a disease characterized by memory loss, executive dysfunction, cognitive disorder and personality changes [1]. It affects about 11 percent of persons over the age of 65 [2] and the prevalence increased by about 161 percent from 1990 to 2019 [3]. The current standard of care to screen for cognitive issues is the Comprehensive Geriatric Assessment, a technique that is difficult to provide at scale [4]; the increasing prevalence of AD has spurred the development of potential easily distributed “digital biomarkers” [5]. It has been well established that speech changes, including impaired semantic processing [6] and reduced lexical diversity [7] are common in the early phases of AD, suggesting that digital analysis of spontaneous speech might be one way to screen for this condition. If a model could be developed with an adequate level of recall/sensitivity to detect spontaneous speech changes in AD, it could be incorporated into an initial screening process–either in primary care (to indicate who needs referral to a specialized memory clinic) or as an initial automated part of the triage process for the specialized clinics themselves.

One prominent approach in the field is to analyze the audio and linguistic data using domain expertise and manually engineer features that can be used for the classification task [8,9]. This approach, however, misses out on the higher-order features present in the data that are not apparent to the human expert but can be found using artificial intelligence (AI) and machine learning (ML) techniques. With the rise of deep learning in recent years, researchers have utilized transformer-based models to convert texts and audio data into corresponding representations for the purpose of employing different classification approaches. Both the ADReSS (Alzheimer’s Dementia Recognition through Spontaneous Speech) and ADReSSo (Alzheimer’s Dementia Recognition through Spontaneous Speech, audio only) challenges at Interspeech 2020 and 2021 facilitated progress in this field by making standardized Alzheimer’s dementia datasets available for researchers to build classification and regression models [10]. Although the dataset has known limitations (poorly characterized and very few audio files from non-English speakers) it is the largest such dataset currently available. Analyses have been conducted as well using support vector machines and artificial neural networks for the task of classification based on grammar based linguistic features, word embeddings generated from Bidirectional Encoder Representations from Transformers (BERT), acoustic features, and wav2vec embeddings [11].

However, there are four major methodological limitations common across this body of work. First, many studies apply a single *a priori* selected ML model to their datasets without sequentially trying a number of models and filtering for those that have the best performance, based on a pre-determined benchmark. Second, there is a pervasive lack of truly independent validation datasets [12]; most studies rely on data partitions (e.g., test sets) derived from the same source as the training data. This can greatly limit the generalizability of their findings to other populations, especially since much of this data was collected between 1983–1988 [13]. Third, most of the collected datasets contain only AD versus control subjects; there is no attempt to validate the use of these models in a real clinical setting where patients are referred with a variety of cognitive issues. Fourth, much of this work trains classification models on manually (human) derived transcripts [12] as opposed to ML transcription models which would be required if this approach was to be used practically to screen patients for dementia. The current study seeks to correct some of these issues using the Pitt dataset [14] which has recognized limitations such size constraints and data imbalances (such as higher prevalence of dementia and more women than men) [14].

The objective of our study was to create a model to screen for Alzheimer’s by implementing a sequential process to systematically evaluate the performance of various techniques and to validate model performance on both a truly independent validation dataset (AD versus control) and a “real world” dataset commonly found in memory clinics.

## Methods

### Ethics statement

Our study was approved by the University of British Columbia’s Committee for the Protection of Human Subjects (approval number H24-01019). All data collected in the DementiaBank repository was collected after obtaining formal consent and all participants provided permission for the reuse of this data and for the placement of their data on an open access repository [15].

### Model development dataset

DementiaBank is an open-access repository of audio “data from communicative interactions from people with dementia, mild cognitive impairment (MCI), and controls” and has greatly facilitated progress in this field by making standardised datasets in English available for researchers to build classification models [15]. We worked with the Pitt dataset [14] (a superset from which the original ADReSS dataset was drawn from for the ADReSS and ADReSSo challenges at Interspeech 2020 and 2021) [16]. This dataset contains spontaneous speech data based on the “Cookie Theft Picture” [17] a standard neuropsychological test of the ability to produce speech. All subjects are classified as AD or control (CN), all subjects have MMSE scores and this dataset has been used for classification studies in the past [18]. The datasets are provided by registration only and the subject pool has been described previously [14].

The Pitt dataset contained 549 audio files which were divided into a training set (337 examples, 187 AD, 150 control), a development set (115 examples, 64 AD, 51 control) and a test set (97 examples, 54 AD, 43 control). When splitting the Pitt dataset, we recognized the need to conduct a stratified split to ensure that each split had similar proportions for the features of diagnosis, biological sex, and a binary variable of age before 65 or not for the first test of a participant. We also recognized that we needed to prevent leakage of data between the splits since a participant can have multiple tests over the period of many years. As a result, each participant can only be in one split. In the end, the train data had 174 participants corresponding to 337 examples, the dev dataset has 59 participants corresponding to 115 examples, and the test dataset has 59 participants corresponding to 97 examples. The Pitt dataset contained 63 percent women with an average age of 67.8 ± 0.5. The average MMSE was 17.8 ± 0.5 in the subjects with AD and 29.0 ± 0.1 in the CN subjects.

### Model validation dataset

A completely independent dataset (also from DementiaBank) [15] was used for the final validation of our top performing model. This dataset (the Lu dataset) [19] contains 27 controls, 16 patients with Alzheimer’s disease, and 11 patients with other diagnoses (1 with vascular dementia, 2 with minimal cognitive impairment, 2 with frontal temporal dementia and 7 with dementia not otherwise specified). Only the controls and AD data were used for the initial validation of the model. The entire dataset was used for the testing of the model on a dataset similar to referrals to a specialized memory clinic, containing a mix of controls, AD and other etiologies of dementia. The validation dataset was 63 percent women with an average age of 79.4 ± 1.3. The Lu dataset did not record MMSE scores.

### Transcriptions of audio data

For transcriptions of the audio data, we used the Whisper model released by OpenAI [20] as used in previous work [21]. We selected 20 audio files from each group and performed a manual human transcription to compare the word error rate between the two groups to ensure that the automated transcription process was not a confounder.

### Overall approach

In this project, we have experimented with many methods to see which approaches produced the best results. After a review of the literature, the study team came up with a list of possible models to sequentially test on our spontaneous speech datasets (listed below). Each model was first tried on our training dataset–if recall for a model was less than 0.75 on our training dataset the approach was abandoned and we moved on to the next model design. We felt that the recall score is particularly critical in medical applications because instances in which individuals with the disease are incorrectly classified as healthy can carry significant clinical and emotional consequences.

Our most successful model was then tested on our validation dataset. Each method will be discussed in more detail in the subsequent subsections. All analyses were done using Python 3.11.3 (scikit-learn 1.5.0, sentence-transformers 2.7.0, bert extractive summarizer 0.10.1, S-Bert 3.0.1, Whisper v3) [22].

a) Classical baseline/Term Frequency-Inverse Document Frequency (Tfidf)

Tfidf stands for”Term frequency, Inverse Document Frequency) and is one of the most commonly used method used for feature extraction [23]. The Tfidf vectorizer will reward more weights to words that are relatively rare and more representative to the document, and lower weights to words that are common, and therefore less representative to the text [23]. Since the text (transcripts) length can be varying between examples in the dataset, we used the Sklearn Tfidf vectorizer to convert them to fixed length vectors of the same length (10,000) which represent each example [24]. After converting the text to vectors or list of numbers, we then trained our classification model using these fixed-length vectors. For baseline models, we used a commonly used ML library called Sk-learn (also called scikit-learn) for Tfidf processing and logistic regression classification [25]. The classical baseline classification model used Tfidf Vectorizer and Sklearn Logistic regression classifier with the default settings.

b) Hybrid approach (Tfidf ± Text Embeddings)

Although Tfidf method works well at detecting important keywords in a piece of text, it is not designed to capture the complex semantic meanings of a text. One of the recent methods to used to extract semantic meaning of a document is using neural models to produce text embeddings. This has been attempted previously using GPT-3, one of the popular Large Language Models to calculate text embeddings for the same task (classification and regression for people with or without Alzheimer’s Disease) [26]. We elected to use Bert Extractive Summarizer [27] to avoid using a closed source model that is updated regularly making it difficult to replicate the results. Also, in a medical context, most institutions would prefer to have models running in a self-contained environment to protect data privacy.

Text transcripts were pre-processed by lowercasing and removing extraneous characters. An extractive BERT-based summarizer was used to generate a condensed representation of each transcript, which was appended to the original text. The combined text was transformed into two feature representations: (i) a high-dimensional sparse TF–IDF vector and (ii) a dense semantic embedding generated using the LaBSE sentence embedding model. These vectors were concatenated to form a joint feature representation. Classification was performed using logistic regression with L2 regularization. This regularization was used to mitigate overfitting in the high-dimensional feature space.

c) Neural Classification

First, we converted the text into fixed-length vectors using SBERT (sentence transformer) with LaBSe embeding model as described in the hybrid model subsection above. Then we classified the text into AD/CN label using Sk-learn MLP (Multi-layer Perceptron) neural classifier. The network comprised three fully connected hidden layers with 500 units each. Training used the L-BFGS optimizer with a maximum of 500 iterations. L2 regularization (weight decay) was applied via the alpha parameter (α = 1e-5) to mitigate overfitting. No dropout layers or early stopping were employed. The learning rate was initialized at 1e-4, and model initialization was fixed using a random seed of 42 to ensure reproducibility [28]*.*

d) SetFit for Efficient Few-Shot Learning

With the recent advancements in fine-tuning transformer models on low-resource datasets, one technique has been recently developed is SetFit [29] which can be used to fine-tune Sentence Transformer models on small datasets. It uses the already-present world and language knowledge of pre-trained Sentence Transformers to learn useful representations from a limited number of labelled examples. Previous work has shown that this type of fine-tuning can even work with 8 or 16 data points per class [29].

e) Zero-shot and Few-Shot Classifications with Large Language Models (LLMs)

With the recent development of LLMs language models we decided to test their zero-shot and few-shot capabilities for our task. Specifically, we utilized the BioMixtral model [30], which is a pre-trained LLM specialized for biomedical and clinical text. In the case of zero-shot classification, we directly use the pre-trained LLM to classify examples without giving it any additional fine-tuning or reference examples. The model is prompted with a description of the task using natural language and then given the examples that we need to classify. It then uses its pre-existing knowledge base to generate the predictions. The few-shot classification involved providing the LLM with a small number of labelled examples (two examples for each class) and using them to guide the model’s predictions.

In both zero-shot and few-shot classifications, the initial prompt given to the model can have a noticeable effect on the quality of the predictions. Our prompt consisted of: ”In this task, your goal is to predict if the speaker has Alzheimer’s disease based on their description of the Cookie Theft picture from the Boston Diagnostic Aphasia Examination. In this test, the participant is shown a picture depicting a boy stealing cookies from a cookie jar while his sister and mother are distracted. The participant is asked to describe the picture in as much detail as possible. Note: This task is critical to me, so I need you to be accurate in your predictions.” The note at the end of the prompt that states,”This task is critical to me, so I need you to be accurate in your predictions” is an engineering trick that seems to improve the accuracy of the final results by a few points [31].

f) Wav2Vec2 Audio Embeddings ± Logistic Regression

The wav2vec2 model has shown significant promise in the field of speech processing by converting raw audio inputs into meaningful embeddings [32]. Utilizing wav2vec for audio embeddings involves leveraging its capability to pre-train on large amounts of unlabeled audio data, capturing a rich representation of speech features. These embeddings were then used as inputs for our classification task.

### Model metrics

We measured model performance using the accuracy, precision, recall, specificity and F1 score. The accuracy score is the overall percentage of how many correct predictions in all predictions, for all labels (AD/CN), defined as Correct Predictions/Total Predictions. Precision is defined as True Positives/ (True Positives + False Positives). Recall is defined as True Positives/ (True Positive + False Negatives) and is the percentage of how many correct positive AD predictions in all actual AD labels. The recall score is particularly critical in medical applications because instances in which individuals with the disease are incorrectly classified as healthy can carry significant clinical and emotional consequences. Specificity is defined as True Negative/ (True negative + False Positives). The FI score is the harmonic mean of precision and recall [33]. Differences between models were descriptive only.

## Results

### Initial exploratory analysis

Top frequent words in the Pitt training set and their counts:

- AD group: [(’uh’, 430), (’cookie’, 177), (’water’, 175), (’sink’, 161), (’cookie jar’, 143), (’stool’, 134), (’boy’, 128), (’dishe’, 127), (’well’, 123), (’mother’, 123), (’oh’, 102), (’see’, 99), (’laughs’, 93), (’floor’, 89), (’girl’, 86), (’window’, 79), (’one’, 76), (’know’, 72), (’um’, 68), (’g’, 67), (’little girl’, 64), (’look’, 62), (’little boy’, 60), (’got’, 56), (’hand’, 52), (’kid’, 51), (’guess’, 48), (’going’, 48), (’xxx’, 47), (’sister’, 47), (’think’, 46), (’dryin g’, 45), (’okay’, 44), (’standing’, 44), (’reaching’, 43), (’g dishes’, 43), (’runnin g’, 43), (’falling’, 41), (’gonna’, 41), (’open’, 39), (’dish’, 37), (’s’, 36), (’want’, 36), (’something’, 35), (’getting’, 35), (’running’, 33), (’drying’, 33), (’yeah’, 33), (’washin g’, 33), (’plate’, 32)]
- CN group: [(’uh’, 345), (’mother’, 176), (’water’, 145), (’cookie’, 145), (’stool’, 144), (’sink’, 139), (’um’, 111), (’boy’, 100), (’dishes’, 99), (’see’, 97), (’window’, 92), (’cookie jar’, 86), (’okay’, 80), (’action’, 73), (’standing’, 70), (’little girl’, 69), (’reaching’, 68), (’floor’, 62), (’girl’, 60), (’look’, 59), (’g’, 57), (’drying’, 57), (’hand’, 57), (’one’, 53), (’curtain’, 53), (’well’, 49), (’open’, 48), (’little boy’, 48), (’running’, 47), (’falling’, 47), (’outside’, 45), (’going’, 44), (’overflowing’, 44), (’sister’, 39), (’oh’, 38), (’plate’, 37), (’know’, 37), (’getting’, 36), (’fall’, 35), (’out of’, 35), (’drying dishes’, 33), (’want’, 32), (’blowing’, 29), (’dish’, 29), (’s’, 27), (’guess’, 27), (’kitchen’, 25), (’xxx’, 25), (’laugh’, 25), (’washing dishes’, 24)]

We observed that the CN group tended to describe the cookie thief picture with more details (such as about the children stealing cookies, the stool is about to fall, the mother washing dishes, and water overflowing from the sink, etc.). However, people in AD group tend to describe the same picture with much less details, using generic and filler words (”uhm”,”somebody”, etc).

When we calculated the word error rate of our Whisper transcriptions (based on our manual human transcriptions) we found no significant difference between the AD and control groups (t = 0.945, p = 0.351).

### Classical, hybrid and neural classification approaches (Table 1 and Table 2)

Overall, only our classical, hybrid and neural classification approaches met our criteria that recall be greater than 0.75 on our training dataset and were tested further on our testing dataset. The accuracy and recall scores for the classical, hybrid and neural classification approaches are shown Table 1; overall the best performing model was the hybrid approach. The hybrid approach improved the accuracy on the test set as compared to the classical approach from 0.85 to 0.92, and the recall improved from 0.85 to 0.93. In addition, the gap between train accuracy scores (0.91) and test scores (0.92) are narrower than the classical model (0.88) and (0.85) respectively suggesting that the hybrid approach was more generalizable. However, there is still a gap between development scores (0.79, 0.77) compared to test scores (0.92, 0.93) in the hybrid model, suggesting there is still a need for more data to improve generalization ability. The neural model clearly overfitted on the train set, with train scores of 1.00 on both accuracy and recall. It did worse on the test set, with accuracy and recall being 0.72 and 0.85 respectively. The neural model performance is lower than the classical Tfidf model, with test scores on accuracy and recall are 0.85 and 0.85 respectively (Table 2).‌‌

**Table 1:** Model performance (accuracy and recall).

Model	Train accuracy	Train recall	Dev accuracy	Dev recall	Test accuracy	Test recall
Classical Tfidf	0.88 ± 0.02	0.86 ± 0.03	0.75 ± 0.04	0.72 ± 0.06	0.85 ± 0.04	0.85 ± 0.05
Hybrid	0.91 ± 0.02	0.90 ± 0.02	0.79 ± 0.04	0.77 ± 0.05	**0.92** ± 0.03	**0.93** ± 0.03
Neural Classifier	1.00 ± 0.00	1.00 ± 0.00	0.73 ± 0.04	0.75 ± 0.05	0.72 ± 0.05	0.85 ± 0.05

Model performance in the training, development (Dev) and testing datasets.

**Table 2:** Model performance (precision and F1).

Model	Train precision	Train F1	Dev precision	Dev F1	Test precision	Test F1
Classical Tfidf	0.92 ± 0.02	0.89 ± 0.02	0.81 ± 0.05	0.76 ± 0.04	0.93 ± 0.05	0.93 ± 0.03
Hybrid	0.93 ± 0.02	0.92 ± 0.02	0.84 ± 0.05	0.80 ± 0.04	**0.92 ± 0.04**	**0.93 ± 0.03**
Neural Classifier	1.00 ± 0.00	1.00 ± 0.00	0.76 ± 0.05	0.75 ± 0.04	0.70 ± 0.06	0.77 ± 0.04

Model performance in the training, development (Dev) and testing datasets.

### SetFit for efficient few-shot learning

We experimented with various subsets of the training data as examples, ranging from as few as ten examples to the entire training set. Despite our efforts, the best-performing SetFit model achieved an accuracy below 50% and an F1 score under 0.5 in the training dataset and this approach was abandoned.

### Zero-shot and few-shot classifications with Large Language Models (LLMs)

In the case of the few-shot classification, we added two examples for each class. The model struggled to accurately classify individuals with AD based on their Cookie Theft transcripts, even when provided with a few labelled examples. Our accuracy in the training dataset with this approach was only 71%, and this approach was abandoned.

### Wav2Vec2 audio embeddings

In this approach, after extracting audio embeddings using wav2vec, we employed a logistic regression model to classify the presence of Alzheimer’s. This approach only gave an accuracy of 60% on the training dataset.

### Validation dataset (AD versus CN)

**Fig 1:**
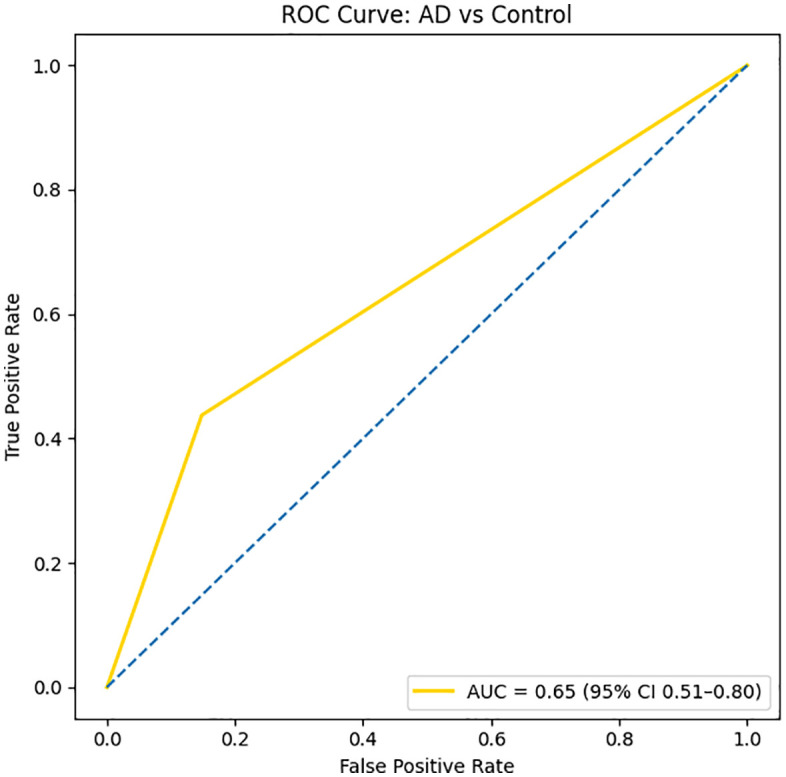
Receiver Operator Curve (true positive rate versus false positive rate) for classification between Alzheimer’s disease and control groups. The model exhibited an accuracy of 0.70 and a recall of 0.44, indicating that model performed well in identifying controls but missed some true AD cases. The area under the curve was 0.64, indicating moderate effectiveness.

Our top performing model (the hybrid approach) was then tested on our independently gathered validation dataset. The model exhibited an accuracy of 0.70 ± 0.07, a recall of 0.44 ± 0.12, a precision of 0.66 ± 0.15, a specificity of 0.86 ± 0.07 and a FI score of 0.53 ± 0.10 on the validation dataset. The area under the curve was 0.64, indicating moderate effectiveness (Fig 1). The model exhibits high precision and specificity, but recall (sensitivity) is somewhat lower indicating that model performed well in identifying controls but missed some true AD cases.

### Undifferentiated dementia validation dataset (undifferentiated dementia versus CN)

**Fig 2:**
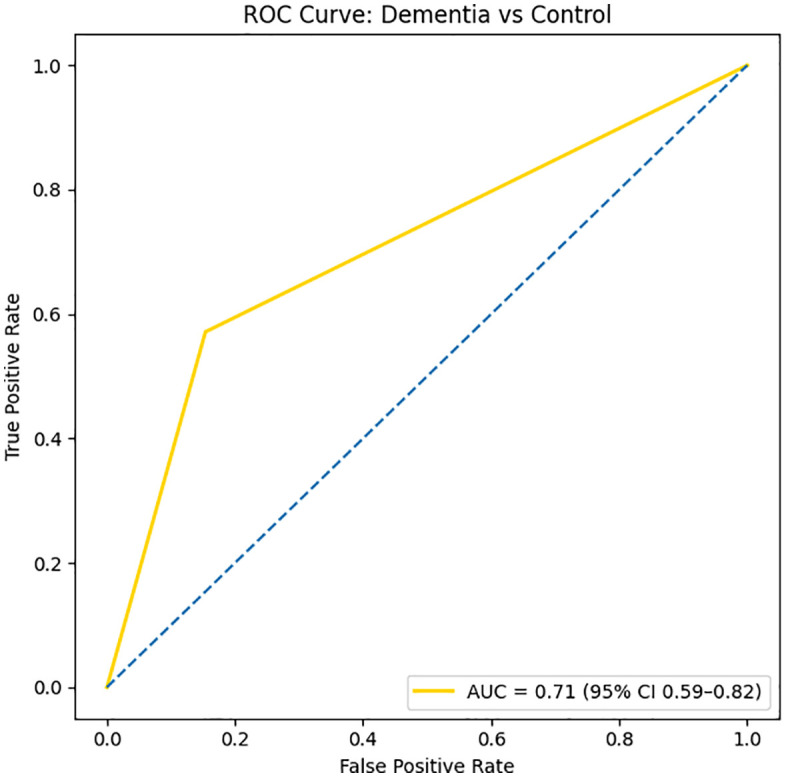
Receiver Operator Curve (true positive rate versus false positive rate) for classification between dementia and control groups. The model exhibited an accuracy of 0.70 and a recall of 0.57, indicating that once again the model performed well in identifying controls but missed some true dementia cases. The area under the curve was 0.71, indicating moderate discriminative power.

In this dataset, the model exhibited an accuracy of 0.70 ± 0.06 and a recall of 0.57 ± 0.12, indicating that once again the model performed well in identifying controls but missed some true dementia cases. The model’s precision was 0.50 ± 0.12, the specificity was 0.76 ± 0.07 and the F1 score was 0.53 ± 0.09. The area under the curve was 0.71, indicating moderate discriminative power (Fig 2).

## Discussion

### Principal findings

We were able to demonstrate, using our Hybrid model (Tfidf + Text Embeddings), some success in classifying audio recordings for the presence or absence of Alzheimer’s disease. When we tested our best model on an independently gathered validation dataset, however, our model only exhibited an accuracy of 0.70 and a recall of 0.44; this drop in performance indicates instability and distribution sensitivity. To our knowledge, both the use of our hybrid approach (Tfidf + Text Embeddings) as well as the use of an independently gathered validation dataset are novel approaches.

### Previous work

To our knowledge, this is the first time various language processing/artificial intelligence techniques have been sequentially tested on their performance on classifying subjects into AD and controls. This is also the first time a hybrid approach (combining Tfidf and BERT summarizer text embeddings) has been examined and tested on a completely independently collected validation dataset.

Previous investigators have used simple BERT text models to approach this classification problem. It is difficult to compare our model’s performance to past approaches without using statistical comparisons (and the datasets were slightly different). Past approaches using BERT text embeddings with held out test set data were done by Guo *et al.* (accuracy of 0.82, n = 156) [34], Haulcy *et al* [35] (accuracy of 0.85, n = 156) and Zhu *et al.* [36] (accuracy of 0.81, n = 156). Other work using BERT text embeddings with a cross-validation approach were studied by Balagopalan *et al.* (accuracy of 0.85, n = 156) [37] and Yuan *et al.* [38] (accuracy of 0.82, n = 156). Past work has also used classical approaches similar to our initial baseline approach, including Millington *et al.* (accuracy of 0.67, n = 156) and Shah *et al* (accuracy of 0.85, n = 156). Unlike our investigation, none of these studies employed a final check against an independently gathered validation dataset.

Our study demonstrated that other methods (such as neural classification, few-shot learning techniques, the use of LLMs and audio embeddings), although more complex, did not attain the accuracy of our hybrid (or even our baseline classical) textual approach. This is congruent with the previous literature where training models on acoustic features [8,39,40] and using neural networks [18] did not attain the accuracy seen in our approach.

Some have tried to train large language models on similar datasets, but these approaches have also demonstrated less accuracy than our hybrid approach [26,41], congruent with our results using zero-shot and few-shot classifications with an LLM. There are a few reasons that can explain the less-than-ideal results of the LLM approach. The main one is that the linguistic patterns associated with AD in the Cookie Theft transcripts may be too subtle and complex for the model to capture without explicit fine-tuning. While extensive, the pre-trained knowledge of BioMixtral (in our paper) may not be sufficient to distinguish between AD and non-AD individuals based solely on a few examples and a task description. This might have been further exacerbated by the possibility that transcriptions in our dataset may be significantly different from the text data used to pre-train BioMixtral (or the datasets used to pretrain the LLMs used in other studies [26,41]). The data used to train LLMs (biomedical research articles and clinical notes in the case of BioMixtral) is likely pretty different from our spontaneous speech dataset; this type of mismatch might have been one factor limiting the model’s ability to use its knowledge to effectively classify subjects into AD and Controls.

Although our hybrid model operated on learned text embeddings as opposed to hand-engineered linguistic features, prior work suggests that persons with Alzheimer’s disease exhibit reduced vocabulary richness, decreased semantic specificity, increased repetition and information impairment [42]. In our exploratory data analysis, we found that persons in the AD group tended to describe the same picture with much less details, using generic and filler words (”uhm”,”somebody”, etc). As well, our hybrid approach which involved the addition of extractive BERT-based text summaries would have likely emphasized any information inaccuracies, contributing to our model’s performance. Future work will need to be done to examine and confirm these speech pattern differences.

### Clinical implications

Although our model demonstrated quite good accuracy (0.92) and recall (0.93) on our test dataset, when we tested it on a completely independently validation dataset, the model performed well in identifying controls but missed quite a few true AD cases. This indicates that although AI approaches to cognitive screening show some promise, caution must be exercised using models in the medical domain since they are often “characterized by limited samples due to the complexity and high costs of patient data collection.” [43] In addition, when tested on the validation dataset, our hybrid model had much poorer recall/sensitivity (0.44) as compared to accuracy (0.70), which raises some concerns about using the model in its current form for clinical screening for AD. Although the models studied in this study are merely at the “proof-of-concept” stage, the success our preliminary hybrid model had in identifying controls, combined with the surprising success in identifying controls in the “real world” dataset suggest that this approach might eventually play a role in triaging patients, as opposed to providing a reliable clinical diagnosis. In theory, patients referred by their family to a specialized memory clinic could undergo initial automated audio screening which would allow more patients with disease to be triaged to be seen more quickly by a physician–this will require more study prior to implementation, however, given the potential differences between our datasets and a typical memory clinic referral population. Since AD is both 1) an important health problem and 2) has effective treatments (cholinesterase inhibitors improve symptoms and slow progression for several years [44] and there are potentially new anti-amyloid therapies on the horizon [45]) AD meets World Health Association guidelines for the development of potential screening tests [46].

Although our results demonstrate possible future utility of our model to screen for AD as a “proof of concept”, prior to any use of our model it will need to be trained on more diverse, multi-center datasets using a simplified model architecture to reduce overfitting on smaller datasets. Also prior to deploying our model to screen for AD, it will likely need to employ lower classification thresholds to increase recall to higher than 0.85, even at the cost of reduced precision/higher false positives. If we tune our current best performing model’s thresholds for a recall of 0.85, our precision drops to only 0.39 on our validation dataset (AD versus Controls) indicating this approach requires further training on much larger and more diverse datasets prior to any clinical implementation.

### Limitations and future research

Given the difficulty in gathering patient data in this population, the DementiaBank data repository is a valuable resource and a valiant attempt to overcome the issue of small datasets in medical AI research [43]. Our best perfoming model (Hybrid approach) showed a large drop in perfomance (both accuracy and recall) when tested on our validation dataset indicating limited generalizability, likely due to a small dataset. Prior to any deployment for AI screening, the model needs to be trained on larger, more diverse multi-centre datasets. Also the subjects that provided these audio files are very poorly characterized, with only age, gender and cognitive scores available. Although DementiaBank is starting to collect audio files from non-English speakers, these datasets are still quite sparse. As these datasets are expanded, potential models could be trained on non-English audio files; this might be a place where the use of LLMs could be more useful, given their ability to easily handle multilingual datasets [47]. Also, the Pitt Dataset had a much higher prevalence of AD than in the general population; this dataset imbalance means that our model would be unsuited for widespread population screening and also the training data might be somewhat different than the population referred to specialized memory clinics. Although outside the scope of the current study, future work using longitudinal data to examine speech changes in those that convert from control status to AD would also be a promising line of investigation. Due to the fact age is a major risk factor for the development of AD, persons with AD tend to be older than controls. As a result, some linguistic differences captured by the model may reflect age-related language changes rather than disease-specific effects.

Our hybrid model used numerical text embeddings rather than individual words, which makes direct word-level explanations difficult. Applying SHapley Additive exPlanations (SHAP) to a simpler word-based model could help identify which words or phrases are most associated with Alzheimer’s disease; although this was outside of the scope of the present study this could be explored in future work.

## Conclusions

Using our novel Hybrid model (TF-IDF + text embeddings), we successfully classified audio recordings for Alzheimer’s disease, however this only achieved a 0.70 accuracy and 0.44 recall on an independent validation dataset.
